# Potential underreporting of treated patients using a *Clostridioides difficile* testing algorithm that screens with a nucleic acid amplification test

**DOI:** 10.1017/ice.2023.262

**Published:** 2024-01-25

**Authors:** Alice Y. Guh, Scott Fridkin, Dana Goodenough, Lisa G. Winston, Helen Johnston, Elizabeth Basiliere, Danyel Olson, Christopher D. Wilson, Jasmine J. Watkins, Lauren Korhonen, Dale N. Gerding

**Affiliations:** 1 Division of Healthcare Quality Promotion, Centers for Disease Control and Prevention, Atlanta, Georgia; 2 Emory University School of Medicine, Atlanta, Georgia; 3 Georgia Emerging Infections Program, Decatur, Georgia; 4 Atlanta Veterans’ Affairs Medical Center, Decatur, Georgia; 5 University of California, San Francisco, School of Medicine, San Francisco, California; 6 Colorado Department of Public Health and Environment, Denver, Colorado; 7 Connecticut Emerging Infections Program, Yale School of Public Health, New Haven, Connecticut; 8 Tennessee Department of Health, Nashville, Tennessee; 9 Edward Hines, Jr., Veterans’ Affairs Hospital, Hines, Illinois

## Abstract

**Objective:**

Patients tested for *Clostridioides difficile* infection (CDI) using a 2-step algorithm with a nucleic acid amplification test (NAAT) followed by toxin assay are not reported to the National Healthcare Safety Network as a laboratory-identified CDI event if they are NAAT positive (+)/toxin negative (−). We compared NAAT+/toxin− and NAAT+/toxin+ patients and identified factors associated with CDI treatment among NAAT+/toxin− patients.

**Design:**

Retrospective observational study.

**Setting:**

The study was conducted across 36 laboratories at 5 Emerging Infections Program sites.

**Patients:**

We defined a CDI case as a positive test detected by this 2-step algorithm during 2018–2020 in a patient aged ≥1 year with no positive test in the previous 8 weeks.

**Methods:**

We used multivariable logistic regression to compare CDI-related complications and recurrence between NAAT+/toxin− and NAAT+/toxin+ cases. We used a mixed-effects logistic model to identify factors associated with treatment in NAAT+/toxin− cases.

**Results:**

Of 1,801 cases, 1,252 were NAAT+/toxin−, and 549 were NAAT+/toxin+. CDI treatment was given to 866 (71.5%) of 1,212 NAAT+/toxin− cases versus 510 (95.9%) of 532 NAAT+/toxin+ cases (*P* < .0001). NAAT+/toxin− status was protective for recurrence (adjusted odds ratio [aOR], 0.65; 95% CI, 0.55–0.77) but not CDI-related complications (aOR, 1.05; 95% CI, 0.87–1.28). Among NAAT+/toxin− cases, white blood cell count ≥15,000/μL (aOR, 1.87; 95% CI, 1.28–2.74), ≥3 unformed stools for ≥1 day (aOR, 1.90; 95% CI, 1.40–2.59), and diagnosis by a laboratory that provided no or neutral interpretive comments (aOR, 3.23; 95% CI, 2.23–4.68) were predictors of CDI treatment.

**Conclusion:**

Use of this 2-step algorithm likely results in underreporting of some NAAT+/toxin− cases with clinically relevant CDI. Disease severity and laboratory interpretive comments influence treatment decisions for NAAT+/toxin− cases.

Laboratory methods for diagnosing *Clostridioides difficile* infection (CDI), a toxin-mediated disease, have evolved in the United States over the past decade. Nucleic acid amplification tests (NAATs), which first became available in the late 2000s, are highly sensitive for toxigenic *C. difficile* since they detect the toxin gene but not the actual toxin.^1^ Early reports of missed CDI diagnosis due to the poor sensitivity of toxin enzyme immunoassays (EIAs) led to increased use of NAATs.^2-5^ From 2011 to 2017, across multiple US sites, the percentage of incident CDI episodes diagnosed by NAAT alone or as the last test of a multistep algorithm increased from 55% to 83%.^6^ However, as facilities switched from toxin EIA to NAAT, CDI incidence rates increased by 43%–67%.^7^ In addition, evidence started emerging that use of NAAT can potentially lead to an overdiagnosis of CDI (ie, colonization instead of active infection), particularly when used indiscriminately without considering other causes of diarrhea.^8-10^ Consequently, a growing number of US facilities have switched to a 2-step algorithm that has been widely adopted in Europe, which consists of an initial screening with NAAT, and if positive, reflexes to a toxin EIA.^11,12^

Almost all US hospitals are required to report CDI as a laboratory-identified (LabID) event to the National Healthcare Safety Network (NHSN), the nation’s largest surveillance system for healthcare-associated infections.^13^ In 2018, the NHSN changed its reporting protocol to allow facilities that use a multistep algorithm to use only the result of the last test for reporting a CDI LabID event.^14^ Thus, facilities using the 2-step algorithm would not report an NAAT positive (+)/toxin negative (−) result (ie, NAAT-positive result followed by a toxin EIA-negative result) to the NHSN, which has led to decreased CDI rates in some facilities.^15^ However, recent studies indicate that some patients with an NAAT+/toxin− result are treated for CDI,^16-23^ suggesting that a portion of unreported NAAT+/toxin− results are considered active infection. To explore this discrepancy, we conducted a multisite analysis of patients tested by this 2-step algorithm to determine whether NAAT+/toxin− patients had similar characteristics and were as likely to receive CDI treatment as NAAT+/toxin+ patients. We also sought to identify factors associated with CDI treatment among NAAT+/toxin− patients.

## Methods

### CDI surveillance and case definition

The CDC Emerging Infections Program (EIP) conducts active laboratory- and population-based CDI surveillance in 10 US sites.^24^ The surveillance protocol underwent ethical review by CDC and EIP sites and either was deemed nonresearch or received an institutional review board approval with a waiver of informed consent.

Laboratories serving the surveillance areas reported all positive *C. difficile* tests to EIP site staff, including positive tests conducted as part of an algorithm in which the final test is negative. As of 2020, 5 EIP sites (ie, California, Colorado, Connecticut, Georgia, and Tennessee) had laboratories that used this 2-step algorithm for CDI diagnosis. One of the EIP sites had laboratories that only performed the 2-step algorithm in hospitalized patients; laboratories in the other 4 EIP sites had no known testing restrictions. For this analysis, an incident CDI case was defined as a positive *C. difficile* molecular or toxin assay detected during 2018–2020 by the 2-step algorithm in a person aged ≥1 year who did not have a positive test in the prior 8 weeks.

### Data collection

**Figure 1.**
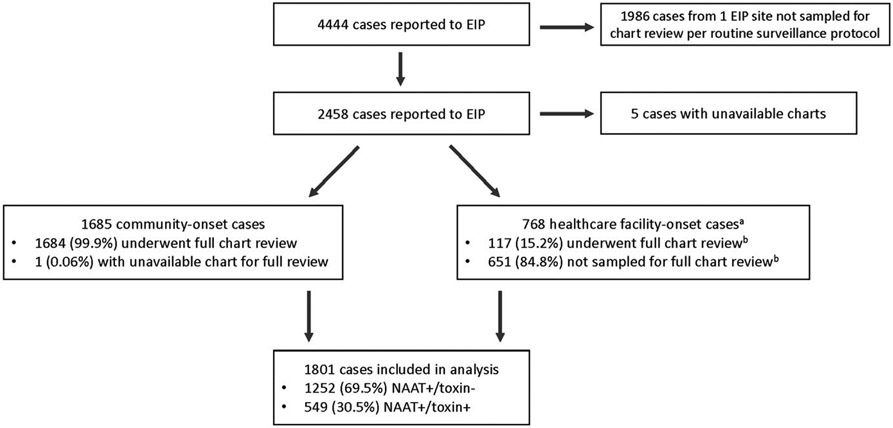
Flow diagram depicting the selection of reported cases for chart review and inclusion in the analysis. Note. EIP, Emerging Infections Program; NAAT, nucleic acid amplification test. ^a^Healthcare facility-onset cases included hospital-onset and long-term care facility onset cases. ^b^The distribution of sampled and non-sampled healthcare facility-onset cases did not differ by sex (52.1% vs 48.9% male; *P* = .51) or age group (61.5% vs 58.1% were aged ≥65 years; *P* = .66), but there were fewer NAAT+/toxin− cases in the sampled group than in the nonsampled group (56.4% vs 69.0%; *P* = .008).

An initial limited chart review was performed on all cases in 4 EIP sites and on a random sample of cases in 1 EIP site (Fig. 1), as previously described.^25^ Based on this review, cases were classified as (1) community-onset if the *C. difficile*–positive stool was collected as an outpatient or within 3 days of hospital admission; (2) hospital-onset if the positive stool was collected >3 days after hospital admission; or (3) long-term care facility (LTCF) onset if the positive stool was collected in an LTCF or from an LTCF resident.

In accordance with the EIP surveillance protocol, all community-onset cases and a random 10%–20% sample of hospital-onset and LTCF-onset cases underwent a subsequent full chart review to collect underlying comorbidities, relevant healthcare and medication exposures, clinical course, and CDI treatment (Fig. 1). Community-onset cases were further classified as community associated if there was no documentation of an overnight stay in a healthcare facility in the preceding 12 weeks. All other community-onset cases were considered healthcare facility associated, and, along with hospital-onset and LTCF-onset cases, were classified as healthcare-associated CDI cases.

Participating laboratories were surveyed annually regarding their *C. difficile* testing method. Laboratories that utilized the 2-step algorithm were asked to share the interpretive comments that they use when reporting out an NAAT+/toxin− result. This information was used to classify laboratories into 2 groups by 3 investigators (A.G., S.F., and D.G.): (1) those that provide no accompanying comments or use neutral wording when reporting an NAAT+/toxin− result (eg, the result could represent colonization or active infection) or (2) those indicating that an NAAT+/toxin− result likely represents *C. difficile* colonization.

### Statistical analysis

Only cases with a full chart review were included in the analysis. Cases were considered as treated for CDI if they were prescribed an appropriate antibiotic treatment according to guidelines^26^ for ≥7 days or were treated until discharge (and had received ≥2 days of treatment), colectomy, or death. Cases who received fecal microbiota transplantation (FMT) were also considered to have been treated for CDI. Comparisons of NAAT+/toxin− versus NAAT+/toxin+ cases and treated versus untreated NAAT+/toxin− cases were described using the χ^2^ and Fisher exact tests (where applicable) for categorical variables and the Wilcoxon rank-sum test for continuous variables. Multiple imputation was performed for the race variable (7.5% of cases were missing) and ethnicity variable (14.2% of cases were missing) using the fully conditional specification method based on age, sex, epidemiologic classification, EIP site, and year.

Separate multivariable logistic regression models were used to compare CDI-related complications (defined as toxic megacolon, ileus, colectomy, or intensive-care unit stay) and CDI recurrence (defined as a *C. difficile*-positive molecular or toxin assay 2–8 weeks following the initial positive test) between NAAT+/toxin− and NAAT+/toxin+ cases. Each model was adjusted for age, sex, race, epidemiologic classification (healthcare-associated versus community-associated CDI), Charlson comorbidity index, and receipt of vancomycin or fidaxomicin within 3 days before or after stool collection. For the outcome of recurrence, we also adjusted for history of CDI in the prior 6 months.

To identify factors associated with CDI treatment among NAAT+/toxin− cases, we used a mixed-effects logistic model adjusting for site clustering (with EIP site as random effect). NAAT+/toxin− cases with missing treatment data or unknown duration of treatment were excluded from the model. The following variables were determined a priori to be potentially associated with treatment and were included in the initial model: age, sex, race/ethnicity, selected comorbidities and healthcare and medication exposures, prior history of CDI, ≥3 unformed stools for ≥1 day, hospital-onset status, hospitalization, white blood cell (WBC) count ≥15,000/μL, and category of laboratory comments regarding an NAAT+/toxin− result. Variables with a *P* value <.10 in the initial model were included in the final model.

Adjusted odds ratios (aOR) and 95% confidence intervals (CI) were calculated for each of the models. A 2-tailed *P* value < .05 was considered statistically significant. SAS version 9.4 statistical software (SAS Institute, Cary, NC) was used for all analyses.

## Results

### Risk factors and clinical characteristics

Of 1,801 cases with a full chart review reported from 36 laboratories, 1,252 (69.5%) were NAAT+/toxin− and 549 (30.5%) were NAAT+/toxin+. The CDI diagnostic assays used by participating laboratories are described in the Supplementary Materials and Supplementary Table S1 (online). A lower percentage of NAAT+/toxin− cases than NAAT+/toxin+ cases were female (57.0% vs 63.9%; *P* = 0.006), non-Hispanic White (48.6% vs 57.9%; *P* = .003), and healthcare associated (37.2% vs 52.1%; *P* < .0001) (Table 1). Median age was lower among NAAT+/toxin− cases than among NAAT+/toxin+ cases (61 vs 67 years; *P* < .0001). NAAT+/toxin− cases were also less likely than NAAT+/toxin+ cases to have been hospitalized (34.1% vs 47.5%; *P* < .0001), to have stayed in an LTCF (3.3% vs 9.8%; *P* < .0001), to have had surgery (9.0% vs 17.5%; *P* < .0001), or to have received antibiotics (63.0% vs 81.2%; *P* < .0001) in the 12 weeks preceding their CDI diagnosis.

Documentation of any diarrhea (≥1 unformed stool) occurred less frequently among NAAT+/toxin− than NAAT+/toxin+ cases: 1,084 (93.8%) of 1,156 versus 506 (97.5%) of 519 (*P* = .001). However, both groups had a similar proportion with ≥3 unformed stools for ≥1 day (49.9% versus 54.1%; *P* = .11). A lower percentage of NAAT+/toxin− cases than NAAT+/toxin+ cases had WBC count ≥15,000/μL (23.9% vs 40.8%; *P* < .0001), serum albumin ≤2.5 g/dL (25.2% vs 31.1%; *P* = .02), pseudomembranous colitis (14.3% vs 40.0%; *P* = .009), and CDI recurrence (7.8% vs 16.6%; *P* < .0001) (Table 1).

In multivariable analysis, NAAT+/toxin− status was protective for CDI recurrence (aOR, 0.65; 95% CI, 0.55–0.77) but not for CDI-related complications (aOR, 1.05; 95% CI, 0.87–1.28) (Table 2).

### CDI treatment

CDI treatment was prescribed to 866 (71.5%) of 1,212 NAAT+/toxin− versus 510 (95.9%) of 532 NAAT+/toxin+ cases (*P* < .0001) (Table 3). Both NAAT+/toxin− and NAAT+/toxin+ cases had the same median duration of treatment of 12 days (IQR, 10–15). A similar proportion of NAAT+/toxin− and NAAT+/toxin+ cases with CDI-related complications received treatment within 48 hours of CDI diagnosis (93.7% vs 100.0%; *P* = .08). Overall, NAAT+/toxin− cases were less likely than NAAT+/toxin+ cases to have received fidaxomicin (2.7% vs 7.1%; *P* < .0001). Documentation of FMT was rare among both NAAT+/toxin− and NAAT+/toxin+ cases (0.8%). Similar results were seen when stratified by hospital-onset status (Table 3).

The percentage of treated NAAT+/toxin− cases per EIP site ranged from 40% to 79%. Laboratories located in the same EIP site often used similar language for reporting an NAAT+/toxin− result (Table S2). As of 2020, of the 36 laboratories, 20 (55.6%) located in 5 EIP sites provided comments indicating that an NAAT+/toxin− result likely represented colonization, and 16 (44.4%) located in four EIP sites provided either no comments (n = 3) or neutral comments (n = 13). NAAT+/toxin− cases reported by the former language were less likely to have received CDI treatment compared to NAAT+/toxin− cases reported by the latter language: 358 (60.7%) of 590 versus 508 (81.7%) of 622 (*P* < .0001). Testing for other enteric pathogens was performed for 611 (49%) of the NAAT+/toxin− cases; of these, 57 (9.3%) tested positive for another enteric pathogen, of whom 36 (63.2%) still received CDI treatment. A comparison of treated and untreated NAAT+/toxin− cases is shown in Table 4.

The initial multivariable model to identify factors associated with CDI treatment in NAAT+/toxin− cases is shown in Supplementary Table S3 (online). In the final multivariable analysis, WBC ≥15,000/μL (aOR, 1.87; 95% CI, 1.28–2.74), ≥3 unformed stools for ≥1 day (aOR, 1.90; 95% CI, 1.40–2.59), and diagnosis by a laboratory that provided no or neutral comments (aOR, 3.23; 95% CI, 2.23–4.68) were significantly associated with receiving CDI treatment among NAAT+/toxin− cases (Table 5). Non-Hispanic, race other than White, compared to non-Hispanic White race (aOR, 0.67; 95% CI, 0.47–0.94), history of CDI in the prior 6 months (aOR, 0.47; 95% CI, 0.26–0.87), and being hospitalized (aOR, 0.54; 95% CI, 0.35–0.86) were associated with no CDI treatment among NAAT+/toxin− cases.

## Discussion

In our large, multisite analysis, NAAT+/toxin− cases were less likely to have traditional CDI risk factors and recurrence but were as likely to have CDI-related complications as NAAT+/toxin+ cases. Notably, although almost all NAAT+/toxin+ cases were treated for CDI, the number of NAAT+/toxin− cases who received CDI treatment was 1.7 times that of NAAT+/toxin+ cases, given the larger number of NAAT+/toxin− cases. A subset of these treated NAAT+/toxin− cases were hospitalized but would not have been reported to the NHSN as a CDI LabID event, indicating that the use of the 2-step algorithm may result in underreporting of clinically relevant CDI.

NAAT+/toxin− patients are more likely to be colonized with *C. difficile*, as indicated by a smaller proportion with traditional CDI risk factors (eg, older age, prior healthcare exposures). However, the findings of pseudomembranous colitis and recurrent CDI among our NAAT+/toxin− cases, albeit at a lower rate than in the NAAT+/toxin+ cases, indicate that a subgroup likely has CDI. Despite having milder disease, NAAT+/toxin− cases had a similar rate of CDI-related complications as NAAT+/toxin+ cases even after adjusting for potential confounders, which is consistent with a previous analysis.^27^ Furthermore, 2 of the NAAT+/toxin− cases required colectomy. Several studies have described similar clinical manifestations and outcomes, including fulminant colitis and colectomy, among a small subset of NAAT+/toxin− patients,^23,27-29^ and as many as 43%–73% of NAATA+/toxin− patients may have probable or possible CDI based on clinical reviews.^18,22,29^

In response to concerns that the current NHSN CDI LabID event definition does not distinguish between colonization and infection and might influence the choice of testing strategy, the NHSN is updating its CDI surveillance definition to include any positive *C. difficile* test and receipt of CDI treatment.^30^ The updated CDI measure, known as healthcare-facility–onset, antibiotic-treated CDI (HT-CDI), has completed validation, and if widely implemented, could improve the reporting of clinically relevant NAAT+/toxin− cases. However, this is predicated on the assumption that clinicians can determine the appropriate NAAT+/toxin− patient to treat, which remains a conundrum.^31^ For example, we found 28.5% of NAAT+/toxin− cases were not treated for CDI, and 8% of the untreated NAAT+/toxin− cases had a subsequent recurrence as defined in this study. Although this finding is similar to the recurrence rate of the treated NAAT+/toxin− cases (Table 2), it suggests that some of the untreated NAAT+/toxin− cases might have had active infection at the time of their initial presentation and would probably have benefited from treatment. In contrast, 71.5% of NAAT+/toxin− cases received CDI treatment, and it is unclear what proportion of these cases might represent overtreatment. Studies that assessed outcomes of untreated NAAT+/toxin− patients have mostly reported no increase in adverse outcomes at 30 days or 8 weeks,^8,20,29^ indicating that most may not need CDI treatment. Although the results of these studies are largely reassuring, a small number of untreated NAAT+/toxin− patients had clinical worsening requiring initiation of treatment and CDI-related ICU admission.^23,29^

Although treatment decisions should be based on individual patient assessment, they are likely influenced by how the test results are presented, such as whether the NAAT+ and toxin− results are reported sequentially or simultaneously and whether they are accompanied by any interpretive comments.^31^ In fact, the absence of any comments or the use of neutral wording to report the NAAT+/toxin− result (eg, the result could represent either colonization or active infection) was significantly associated with receiving CDI treatment. Conversely, NAAT+/toxin− cases were significantly less likely to be treated if there was wording indicating that the patient may be colonized. This finding is consistent with a study that found that switching from NAAT testing to the 2-step algorithm and reporting an NAAT+/toxin− result as “likely colonized” was associated with a decreased likelihood of CDI treatment.^17^

Not surprisingly, having leukocytosis and ≥3 unformed stools for ≥1 day were significant predictors of CDI treatment in NAAT+/toxin− cases. In contrast, having CDI in the prior 6 months was associated with less CDI treatment, which was unexpected, given that risk of CDI increases after a previous episode. It is not known, however, what proportion of the prior CDI episodes were not known to the clinician or were also NAAT+/toxin− and may have been considered colonization. Interestingly, being hospitalized was also protective against treatment. This finding might reflect greater access to antimicrobial stewardship experts and other specialists who may have a higher threshold for treating NAAT+/toxin− patients. Similarly, in another study, infectious diseases consultations were associated with not prescribing CDI treatment to NAAT+/toxin− patients.^23^

Our analysis had several limitations. Because only a sample of hospital-onset cases underwent full chart review and could be included in our analysis, our data might not be representative of hospital-onset cases, which are targeted by pay-for-performance programs. However, sampling was performed randomly to minimize bias. Although this large, multisite analysis was conducted across diverse geographical areas, our findings might not be representative of all facilities reporting to the NHSN. Relevant exposures and treatment data could have been underestimated if there was incomplete documentation in patient charts or if parts of the chart were unavailable for review. In addition, relevant imaging studies and laboratory work were not performed for every patient, especially outpatients, which may have limited our ability to assess CDI-related complications and disease severity. We did not systematically capture positive *C. difficile* tests of patients who sought care outside the surveillance catchment areas, which could have underestimated prior and recurrent CDI rates. Recurrence could have also been underestimated because it relies on clinical practice of test ordering, and intensity of testing diarrheal stools has been shown to vary between institutions.^32^ We also did not collect information on whether there were dedicated antimicrobial stewardship staff reviewing every NAAT+/toxin− result, which could have influenced treatment decisions. Lastly, we only evaluated the use of NAAT as a screening test, and we did not assess the potential use of glutamate dehydrogenase (GDH)-based screening. However, our findings are similar to those of a prior analysis that evaluated an algorithm utilizing GDH/toxin EIA with reflex to NAAT for discrepant results.^27^

In conclusion, the 2-step algorithm can result in substantial underreporting to the NHSN of treated NAAT+/toxin− patients, some of whom may have active infection. The interpretive comments used for presenting an NAAT+/toxin− result can be helpful in reducing unnecessary CDI treatment, but if the language biases the clinician to view every NAAT+/toxin− patient as colonized, the comments could prevent CDI treatment in a subset of patients who might benefit. Furthermore, although the new NHSN CDI metric is intended to better capture clinically relevant CDI, it could potentially tip the scales toward undertreatment of NAAT+/toxin− patients in facilities that are attempting to lower their reported CDI rates. Thus, it remains critical that treatment decisions should be driven by patient’s clinical presentation and risk factors rather than by the type of test or metric used. Further research is needed to determine which NAAT+/toxin− patients are likely to benefit from CDI treatment. This research should build upon the efforts of a small number of observational studies that have attempted to identify predictors of CDI-related complications to help inform treatment decisions,^23,28^ as well as advancing the research in CDI diagnostic assays to accurately distinguish true infection from colonization.

## Supplementary Material

Supplementary Material

## Figures and Tables

**Table 1.** Comparison of Risk Factors and Clinical Characteristics Between NAAT+/toxin− and NAAT+/toxin+ *Clostridioides difficile* Infection Cases

Variable	Overall (N = 1,801), No. (%)^a^	NAAT+/Toxin− (n = 1,252), No. (%)^a^	NAAT+/Toxin+ (n = 549), No. (%)^a^	*P* Value
**EIP site**				.04
California	145 (8.1)	105 (8.4)	40 (7.3)	
Colorado	270 (15.0)	197 (15.7)	73 (13.3)	
Connecticut	110 (6.1)	63 (5.0)	47 (8.6)	
Georgia	799 (44.4)	551 (44.0)	248 (45.2)	
Tennessee	477 (26.5)	336 (26.8)	141 (25.7)	
**Demographic**				
Sex, female	1,065 (59.1)	714 (57.0)	351 (63.9)	.006
Race/ethnicity				.003
Hispanic, any race	104 (5.8)	77 (6.2)	27 (4.9)	
Non-Hispanic, White race	927 (51.5)	609 (48.6)	318 (57.9)	
Non-Hispanic, other race	493 (27.4)	368 (29.4)	125 (22.8)	
Unknown	277 (15.4)	198 (15.8)	79 (14.4)	
Age, median y (IQR)	63 (45–74)	61 (43–72)	67 (51–78)	<.0001
**Epidemiologic classification**				<.0001
Community associated	1,049 (58.3)	786 (62.8)	263 (47.9)	
Healthcare associated	752 (41.8)	466 (37.2)	286 (52.1)	
Community-onset healthcare facility-associated	635 (35.3)	400 (32.0)	235 (42.8)	
Hospital onset	95 (5.3)	58 (4.6)	37 (6.7)	
Long-term care facility onset	22 (1.2)	8 (0.6)	14 (2.6)	
**Charlson comorbidity index**				.42
0	592/1,797 (32.9)	410/1,248 (32.9)	182 (33.2)	
1	292/1,797 (16.3)	212/1,248 (17.0)	80 (14.6)	
≥2	913/1,797 (50.8)	626/1,248 (50.2)	287 (52.3)	
**Prior healthcare exposures** ^b^				
Hospitalization	686/1,795 (38.2)	426/1,248 (34.1)	260/547 (47.5)	<.0001
LTACH stay	10/1,799 (0.6)	4/1,250 (0.3)	6 (1.1)	.08
Long-term care facility stay	95/1,799 (5.3)	41/1,250 (3.3)	54 (9.8)	<.0001
Emergency room visit	498/1,796 (27.7)	345/1,248 (27.6)	153/548 (27.9)	.90
Observational unit stay	74/1,796 (4.1)	44/1,248 (3.5)	30/548 (5.5)	.06
Chronic hemodialysis	154/1,799 (8.6)	120/1,250 (9.6)	34 (6.2)	.02
Surgery	208/1,797 (11.6)	112/1,248 (9.0)	96 (17.5)	<.0001
**Prior medication exposures** ^b^				
Antibiotic	1,226/1,789 (68.5)	782/1,242 (63.0)	444/547 (81.2)	<.0001
Proton pump inhibitor	1,127/1,793 (62.9)	773/1,247 (62.0)	354/546 (64.8)	.25
Immunosuppressant	1,185/1,794 (66.1)	831/1,247 (66.6)	354/547 (64.7)	.43
**Clinical course and outcome**				
≥3 unformed stools for ≥1 day	858/1,675 (51.2)	577/1,156 (49.9)	281/519 (54.1)	.11
Hospital admission^c^	1,335/1,800 (74.2)	947/1,251 (75.7)	388 (70.7)	.02
ICU admission^d^	119/1,800 (6.6)	86/1,251 (6.9)	33 (6.0)	.50
WBC ≥15,000/μL	464/1,604 (28.9)	269/1,126 (23.9)	195/478 (40.8)	<.0001
Serum albumin ≤2.5 g/dL	403/1,494 (27.0)	266/1,054 (25.2)	137/440 (31.1)	.02
Pseudomembranous colitis	20/104 (19.2)	12/84 (14.3)	8/20 (40.0)	.009
Toxic megacolon or ileus	55/979 (5.6)	37/678 (5.5)	18/301 (6.0)	.74
Colectomy	3/1,800 (0.2)	2/1,251 (0.2)	1/549 (0.2)	1.00
CDI-related complications^e^	162/1,002 (16.2)	113/695 (16.3)	49/307 (16.0)	.91
CDI recurrence^f^	189 (10.5)	98 (7.8)	91 (16.6)	<.0001

Note. EIP, Emerging Infections Program; NAAT, nucleic acid amplification test; IQR, interquartile range; LTACH, long-term acute-care hospital; ICU, intensive care unit; WBC, white blood cell; CDI, *Clostridioides difficile* infection.

a Data are presented as no. (%) unless otherwise specified. Any missing response to a variable is excluded from the denominator.

b During the 12 weeks prior to CDI diagnosis.

c Hospitalized at the time of or within 6 d following CDI diagnosis.

d Admitted to the ICU on the day of or within 6 d following CDI diagnosis.

e CDI-related complications defined as toxic megacolon, ileus, colectomy, or ICU admission on the day of or within 6 d following CDI diagnosis.

f CDI recurrence defined as a *C. difficile*-positive stool 2–8 weeks following the initial positive test.

**Table 2.** Multivariable Models Assessing *Clostridioides difficile* Infection (CDI)– Related Complications and Recurrence Between NAAT+/toxin− and NAAT+/toxin+ CDI Cases

	NAAT+/Toxin− vs NAAT+/Toxin+ CDI Cases	
Outcome	Adjusted Odds Ratio (95% CI)	*P* Value
CDI-related complications^a^	1.05 (0.87–1.28)	.60
CDI recurrence^b^	0.65 (0.55–0.77)	<.0001

Note. NAAT, nucleic acid amplification test; CDI, *Clostridioides difficile* infection; CI, confidence interval.

a CDI-related complications defined as toxic megacolon, ileus, colectomy, or intensive-care unit admission on the day of or within 6 d following CDI diagnosis.

b CDI recurrence defined as a *C. difficile*-positive stool 2–8 weeks following the initial positive test.

**Table 3.** Comparison of Treatment for *Clostridioides difficile* Infection (CDI) Between NAAT+/Toxin− and NAAT+/Toxin+ CDI Cases

Variable	Overall^a^	NAAT+/Toxin−^a^	NAAT+/Toxin+^a^	*P* Value
**All cases** ^b^	N=1,744	n=1,212	n=532	
Received CDI treatment	1376 (78.9)	866 (71.5)	510 (95.9)	<.0001
Any oral or rectal vancomycin	1,230/1,376 (89.4)	774/866 (89.4)	456/510 (89.4)	.98
Any fidaxomicin	59/1,376 (4.3)	23/866 (2.7)	36/510 (7.1)	<.0001
Metronidazole only	129/1,376 (9.4)	85/866 (9.8)	44/510 (8.6)	.47
FMT	11/1,376 (0.8)	7/866 (0.8)	4/510 (0.8)	1.00
**Hospital-onset cases** ^c^	N=93	n=57	n=36	
Received CDI treatment	74 (79.6)	40 (70.2)	34 (94.4)	.007
Any oral or rectal vancomycin	70/74 (94.6)	38/40 (95.0)	32/34 (94.1)	1.00
Any fidaxomicin	5/74 (6.8)	0/40 (0)	5/34 (14.7)	.02
Metronidazole only	3/74 (4.1)	2/40 (5.0)	1/34 (2.9)	1.00
FMT	0/74 (0)	0/40 (0)	0/34 (0)	…
**Non-hospital onset cases** ^d,e^	N=1651	n=1155	n=496	
Received CDI treatment	1,302 (78.9)	826 (71.5)	476 (96.0)	<.0001
Any oral or rectal vancomycin	1,160/1,302 (89.1)	736/826 (89.1)	424/476 (89.1)	.99
Any fidaxomicin	54/1,302 (4.2)	23/826 (2.8)	31/476 (6.5)	.001
Metronidazole only	126/1,302 (9.7)	83/826 (10.1)	43/476 (9.0)	.55
FMT	11/1,302 (0.8)	7/826 (0.9)	4/476 (0.8)	1.00

Note.NAAT, nucleic acid amplification test; FMT, fecal microbiota transplantation.

a Data are presented as no. (%). Any missing response to a variable is excluded from the denominator.

b Excludes 57 cases (40 NAAT+/toxin− cases and 17 NAAT+/toxin+ cases) with missing CDI treatment data or unknown duration of CDI treatment.

c Excludes 2 cases (1 NAAT+/toxin− case and 1 NAAT+/toxin+ case) with missing CDI treatment data or unknown duration of CDI treatment.

d Excludes 55 cases (39 NAAT+/toxin− cases and 16 NAAT+/toxin+ cases) with missing CDI treatment data or unknown duration of CDI treatment.

e Non–hospital-onset cases include community-associated CDI cases, community-onset healthcare facility-associated CDI cases, and long-term care facility-onset CDI cases.

**Table 4.** Characteristics of NAAT+/Toxin− Cases by *Clostridioides difficile* infection Treatment Status^a^

Variable	Treated for CDI (N = 866), No. (%)^b^	Not treated for CDI (N = 346), No. (%)^b^	*P* Value
**Demographic**			
Sex, female	501 (57.9)	186 (53.8)	.19
**Race/ethnicity**			.02
Hispanic, any race	48 (5.5)	26 (7.5)	
Non-Hispanic, White race	436 (50.4)	154 (44.5)	
Non-Hispanic, other race	239 (27.6)	121 (35.0)	
Unknown	143 (16.5)	45 (13.0)	
Age, median y (IQR)	62 (47–73)	57.5 (37–70)	.0001
**Select medical conditions**			
Chronic liver disease	42/864 (4.9)	29 (8.4)	.02
Chronic kidney disease	223/864 (25.8)	84 (24.3)	.58
Diabetes mellitus	269/864 (31.1)	107 (30.9)	.94
Diverticular disease	94/864 (10.9)	33 (9.5)	.49
Hematologic or solid-tumor malignancy	148/864 (17.1)	55 (15.9)	.60
Hematopoietic stem-cell or solid-organ transplant	37/864 (4.3)	16 (4.6)	.79
Inflammatory bowel disease	72/864 (8.3)	36 (10.4)	.25
History of CDI in prior 6 mo	41 (4.7)	29 (8.4)	.01
**Prior healthcare exposures** ^c^			
Hospitalization	285 (32.9)	130/344 (37.8)	.11
Long-term acute-care hospital stay	4 (0.5)	0/345 (0)	.58
Long-term care facility stay	31 (3.6)	9/345 (2.6)	.39
Emergency room visit	243 (28.1)	96/344 (27.9)	.96
Observational unit stay	28 (3.2)	16/344 (4.7)	.23
Chronic hemodialysis	81 (9.4)	38/345 (11.0)	.38
Surgery	73 (8.4)	36 (10.5)	.26
**Prior medication exposures** ^c^			
Antibiotic	543/862 (63.0)	217 (62.7)	.93
Proton pump inhibitor	322 (37.2)	140 (40.5)	.29
Immunosuppressant	276 (31.9)	128 (37.0)	.09
**Clinical course and outcome**			
≥3 unformed stools for ≥1 d	443/825 (53.7)	126/297 (42.4)	.0009
Hospital admission^d^	658/865 (76.1)	275 (79.5)	.20
ICU admission^e^	62 (7.2)	22 (6.4)	.62
WBC ≥15,000/μL	205/796 (25.8)	59/310 (19.0)	.02
Serum albumin ≤2.5 g/dL	178/742 (24.0)	83/292 (28.4)	.14
Pseudomembranous colitis	9/53 (17.0)	2/29 (6.9)	.31
Toxic megacolon or ileus	29/502 (5.8)	8/166 (4.8)	.64
Colectomy	2/865 (0.2)	0 (0)	1.00
CDI-related complications^f^	83/513 (16.2)	28/172 (16.3)	.98
CDI recurrence^g^	70 (8.1)	27 (7.8)	.87

Note. NAAT, nucleic acid amplification test; IQR, interquartile range; CDI, *Clostridioides difficile* infection; ICU, intensive care unit; WBC, white blood cell.

a Excludes 40 NAAT+/toxin− cases with missing CDI treatment data or unknown duration of CDI treatment.

b Data are presented as no. (%) unless otherwise specified. Any missing response to a variable is excluded from the denominator.

c During the 12 weeks prior to CDI diagnosis.

d Hospitalized at the time of or within 6 d following CDI diagnosis.

e Admitted to the ICU on the day of or within 6 d following CDI diagnosis.

f CDI-related complications defined as toxic megacolon, ileus, colectomy, or ICU admission on the day of or within 6 days following CDI diagnosis.

g CDI recurrence defined as a *C. difficile*–positive stool 2–8 weeks following the initial positive test.

**Table 5.** Final Multivariable Model to Identify Factors Associated with Treatment for *Clostridioides difficile* Infection Among NAAT+/Toxin− Cases

Variables	Adjusted Odds Ratio (95% CI)	*P* Value
**Race/ethnicity**		
Hispanic, Any race	0.86 (0.48–1.56)	.62
Non-Hispanic, White race	Referent	
Non-Hispanic, Other race	0.67 (0.47–0.94)	.02
History of CDI in prior 6 mo	0.47 (0.26–0.87)	.02
Long-term care facility stay in prior 12 weeks	2.65 (0.97–7.22)	.06
Diagnosis by a laboratory that provided no or neutral comments	3.23 (2.23–4.68)	<.0001
≥3 unformed stools for ≥1 d	1.90 (1.40–2.59)	<.0001
Hospital admission^a^	0.54 (0.35–0.86)	.009
WBC ≥15,000/μL	1.87 (1.28–2.74)	.001

Note. NAAT, nucleic acid amplification test; CDI, *Clostridioides difficile* infection; WBC, white blood cell.

a Hospitalized at the time of or within 6 d following CDI diagnosis.
