# Supplementary material for: Potential underreporting of treated patients using a Clostridioides difficile testing algorithm that screens with a nucleic acid amplification test
Source: Infect Control Hosp Epidemiol. Author manuscript; Available in PMC 2024 May 1. (PMC11027077; doi:10.1017/ice.2023.262)
Supplement: Supplementary Material [file NIHMS1962216-supplement-Supplementary_Material.docx]

**Supplemental Material for “Potential underreporting of treated patients using a *Clostridioides difficile* testing algorithm that screens with a nucleic acid amplification test”**

Results

The following types of NAAT were used for CDI diagnosis by the 36 laboratories included in this analysis: Cepheid Xpert® *C. difficile* (n=32), Meridian Bioscience™ illumigene™ *C. difficile* Test Kit (n=1), and Simplexa® *C. difficile* Direct (n=1). Two laboratories did not provide information on the type of NAAT that they utilized. There were no differences in the distribution of NAAT+/toxin- and NAAT+/toxin+ by NAAT type (*P*=0.10 overall).

The following toxin assays were used for CDI diagnosis by the 36 participating laboratories: Wampole® Tox A/B Quik Chek (n=12), Meridian Bioscience Premier® Toxins A&B (n=9), Techlab Tox A/B Quik Chek® (n=5), Techlab® *C. difficile* Tox A/B II™ (n=3), and Meridian Bioscience Immunocard® Toxins A&B (n=3). Four laboratories did not provide information on the type of toxin assay that they utilized. Excluding these four laboratories with unknown toxin assay, we found there were differences in the distribution of NAAT+/toxin- and NAAT+/toxin+ cases among the different toxin assays utilized by the participating laboratories (*P*=0.004 overall) (Table S1).

**Table S1. Distribution of NAAT+/toxin- and NAAT+/toxin+ *Clostridioides difficile* infection cases by type of toxin assay utilized by participating laboratories^a^**

|  | Wampole® Tox A/B Quik Chek N=489 | Meridian Bioscience Premier® Toxins A&B N=450 | Techlab Tox A/B Quik Chek® N=129 | Techlab® *C. difficile* Tox A/B II™ N=194 | Meridian Bioscience Immunocard® Toxins A&B N=68 | ***P-*value overall** |
| --- | --- | --- | --- | --- | --- | --- |
| **Toxin-positivity status** |  |  |  |  |  | 0.004 |
| NAAT+/toxin- | 312 (63.8) | 339 (75.3) | 85 (65.9) | 138 (71.1) | 46 (67.7) |  |
| NAAT+/toxin+ | 177 (36.2) | 111 (24.7) | 44 (34.1) | 56 (28.9) | 22 (32.4) |  |

Data are presented as no. (%). Abbreviations: NAAT, nucleic acid amplification test.

^a^Excludes cases from four laboratories that did not provide information on the type of toxin assay that they utilized.

**Table S2. Classification of laboratories according to their comments regarding a NAAT+/toxin- test result in 2020, Emerging Infections Program (n=36)**

|  |  |  | NAAT+/toxin- result | |
| --- | --- | --- | --- | --- |
| State | Lab | Laboratory comments | Likely represents colonization | No or neutral comments |
| A | 1 | PCR+/EIA Tox- patients may be colonized. Treatment of these patients may not be necessary, but Isolation Precautions are required. Clinical correlation is required. | X |  |
| A | 2 | No comments |  | X |
| A | 3 | No comments |  | X |
| A | 4 | PCR+/EIA Tox- patients may be colonized. Treatment of these patients may not be necessary, but Isolation Precautions are required. Clinical correlation is required. | X |  |
| A | 5 | PCR+/EIA Tox- patients may be colonized. Treatment of these patients may not be necessary, but Isolation Precautions are required. Clinical correlation is required. | X |  |
| A | 6 | If the EIA is positive, the comment states that there is an active infection. If the EIA is negative, the comment states that the patient is colonized. Both situations specify need for isolation | X |  |
| A | 7 | A negative *C. difficile* toxin immunoassay in the setting of a positive PCR makes C. difficile infection unlikely. Clinical correlation is required. | X |  |
| A | 8 | PCR+/EIA+ patients are considered to have ACTIVE INFECTION. Treatment is recommended based on severity of illness. Isolation Precautions are required. PCR+/ EIA TOX- patients may be COLONIZED. Treatment of these patients may not be necessary, but Isolation Precautions are required. Clinical correlation is required. | X |  |
| A | 9 | PCR+/EIA Tox- patients may be colonized. Treatment of these patients may not be necessary, but Isolation Precautions are required. Clinical correlation is required. | X |  |
| B | 10 | No comments |  | X |
| B | 11 | PCR does not distinguish between colonization and infection and therefore the patient should not be treated until results of the GDH and Toxin A/B assay are obtained. PCR positive GDH positive Toxin negative probable carrier of *C.difficile*, infection unlikely. PCR positive GDH positive Toxin positive, toxigenic *C .difficile* detected, probable infection. | X |  |
| B | 12 | The patient tested positive for *C. difficile* by PCR. This positive result is indicative of colonization but not necessarily infection. If *C. difficile* is suspected as a cause of illness, a C*. difficile* toxin test by EIA is recommended for confirmation. | X |  |
| B | 13 | Positive PCR may represent colonization, infection must be confirmed with GDH/EIA Antigen/Toxin test. Confirmatory testing will be reported within 24 hours of PCR. | X |  |
| C | 14 | Positive PCR and Negative C Diff Toxin most commonly represents a carrier state. This cannot completely rule out active infection. Clinical correlation recommended. If positive PCR and positive EIA: "Positive PCR and Positive C Diff Toxin represents active infection." | X |  |
| C | 15 | PCR+/EIA+: Results suggestive of *C. difficile* infection. PCR+/EIA-: This may represent a *C. difficile* colonization or true infection. Clinical correlation required. |  | X |
| C | 16 | Positive PCR and Negative C Diff Toxin most commonly represents a carrier state. This cannot completely rule out active infection. Clinical correlation recommended. If positive PCR and positive EIA: "Positive PCR and Positive C Diff Toxin represents active infection." | X |  |
| C | 17 | PCR+/EIA-, this may represent a *C. difficile* colonization or true infection. Clinical correlation required. If EIA positive: Results suggestive of *C. difficile* infection. |  | X |
| C | 18 | If positive PCR, negative EIA: "Positive PCR and Negative C Diff Toxin most commonly represents a carrier state. This cannot completely rule out active infection. Clinical correlation recommended." If positive PCR and positive EIA: "Positive PCR and Positive C Diff Toxin represents active infection." | X |  |
| C | 19 | Discordant results, PCR-positive/toxin-negative, may represent colonization (carrier) or a true infection. Clinical correlation is highly advised and consider an infectious disease consult if needed. |  | X |
| C | 20 | Positive PCR and Negative C Diff Toxin most commonly represents a carrier state. This cannot completely rule out active infection. Clinical correlation recommended. If positive PCR and positive EIA: "Positive PCR and Positive C Diff Toxin represents active infection." | X |  |
| C | 21 | Detection of C. diff by PCR may indicate active disease but also may be seen in colonized, asymptomatic individuals. Subsequent detection of C. diff toxin by EIA indicates a high probability of active disease. In toxin negative PCR positive symptomatic patients, clinical correlation is required to determine if treatment is necessary. |  | X |
| C | 22 | If positive PCR, negative EIA: "Positive PCR and Negative C Diff Toxin most commonly represents a carrier state. This cannot completely rule out active infection. Clinical correlation recommended." If positive PCR and positive EIA: "Positive PCR and Positive C Diff Toxin represents active infection." | X |  |
| C | 23 | Detection of C. diff by PCR may indicate active disease but also may be seen in colonized, asymptomatic individuals. Subsequent detection of C. diff toxin by EIA indicates a high probability of active disease. In toxin negative PCR positive symptomatic patients, clinical correlation is required to determine if treatment is necessary. |  | X |
| C | 24 | Detection of C. diff by PCR may indicate active disease but also may be seen in colonized, asymptomatic individuals. Subsequent detection of C. diff toxin by EIA indicates a high probability of active disease. In toxin negative PCR positive symptomatic patients, clinical correlation is required to determine if treatment is necessary. |  | X |
| C | 25 | PCR+/EIA+: Results suggestive of C. difficile infection. PCR+/EIA-: This may represent a C. difficile colonization or true infection. Clinical correlation required. |  | X |
| C | 26 | A positive C. diff Toxin B gene does not indicate a toxin producing C. diff infection. Please refer to the C. diff Toxin EIA test for toxin producing C. diff. Gene B Toxin PCR positive, EIA Toxin Positive=Toxin producing C. diff. Gene B Toxin PCR positive, EIA Toxin Negative=Non Toxin producing C. diff. This test is to be used as an aid in the diagnosis of C. difficile disease. As with other C. difficile tests, results should be considered in conjunction with the patient history. | X |  |
| C | 27 | If positive PCR, negative EIA: "Positive PCR and Negative C Diff Toxin most commonly represents a carrier state. This cannot completely rule out active infection. Clinical correlation recommended." If positive PCR and positive EIA: "Positive PCR and Positive C Diff Toxin represents active infection." | X |  |
| C | 28 | Detection of C. diff by PCR may indicate active disease but also may be seen in colonized, asymptomatic individuals. Subsequent detection of C. diff toxin by EIA indicates a high probability of active disease. In toxin negative PCR positive symptomatic patients, clinical correlation is required to determine if treatment is necessary |  | X |
| C | 29 | PCR+/EIA+: Results suggestive of *C.difficile* infection. PCR+/EIA-: This may represent a *C.difficile* colonization or true infection. Clinical correlation required. |  | X |
| C | 30 | Detection of C. diff by PCR may indicate active disease but also may be seen in colonized, asymptomatic individuals. Subsequent detection of C. diff toxin by EIA indicates a high probability of active disease. In toxin negative PCR positive symptomatic patients, clinical correlation is required to determine if treatment is necessary |  | X |
| C | 31 | If positive PCR, negative EIA: "Positive PCR and Negative C Diff Toxin most commonly represents a carrier state. This cannot completely rule out active infection. Clinical correlation recommended." If positive PCR and positive EIA: "Positive PCR and Positive C Diff Toxin represents active infection." | X |  |
| D | 32 | Detection of bacteria that have the toxin gene but do not produce toxin protein likely reflects colonization with toxigenic *C. difficile* instead of clinical infection. | X |  |
| E | 33 | Discordant results, PCR-positive/toxin negative, may represent colonization (carrier) or a true infection. Clinical correlation is highly advised and consider an Infectious Diseases consult if needed. |  | X |
| E | 34 | Discordant results, PCR-positive/toxin negative, may represent colonization (carrier) or a true infection. Clinical correlation is highly advised and consider an Infectious Diseases consult if needed. |  | X |
| E | 35 | Discordant results, PCR-positive/toxin negative, may represent colonization (carrier) or a true infection. Clinical correlation is highly advised and consider an Infectious Diseases consult if needed. |  | X |
| E | 36 | Toxin- “Toxigenic C. difficile DNA detected BUT free toxin was not detected. These results are suggestive of *C. difficile* colonization and may not reflect *C. difficile* infection requiring treatment. The significance of these results must be interpreted in the context of the patient's clinical scenario.” | X |  |

Abbreviations: NAAT, nucleic acid amplification test; EIA, enzyme immunoassay; GDH, glutamate dehydrogenase

**Table S3. Initial multivariable model to identify factors associated with treatment for *Clostridioides difficile* infection among NAAT+/toxin- cases**

| **Variables** | **Adjusted odds ratio (95% CI)** | ***P*-value** |
| --- | --- | --- |
| **Demographic** |  |  |
| Male sex | 0.96 (0.70–1.31) | 0.78 |
| Age ≥65 years | 1.12 (0.80–1.57) | 0.50 |
| Race/ethnicity |  |  |
| Hispanic, Any race | 0.87 (0.47–1.63) | 0.67 |
| Non-Hispanic, White race | Referent |  |
| Non-Hispanic, Other race | 0.67 (0.46–0.98) | 0.04 |
| **Select medical conditions** |  |  |
| Chronic liver disease | 0.71 (0.38–1.30) | 0.26 |
| Chronic kidney disease | 1.40 (0.87–2.24) | 0.16 |
| Diabetes mellitus | 0.93 (0.66–1.31) | 0.69 |
| Diverticular disease | 1.28 (0.75–2.18) | 0.38 |
| Hematologic or solid tumor malignancy | 1.13 (0.74–1.75) | 0.57 |
| Hematopoietic stem cell or solid organ   transplant | 1.29 (0.59–2.81) | 0.52 |
| Inflammatory bowel disease | 1.14 (0.64–2.01) | 0.65 |
| History of CDI in prior 6 months | 0.48 (0.25–0.90) | 0.02 |
| **Prior healthcare exposures^a^** |  |  |
| Hospitalization | 1.03 (0.72–1.47) | 0.88 |
| Long-term care facility stay | 2.74 (0.99–7.63) | 0.05 |
| Emergency room visit | 1.29 (0.90–1.84) | 0.16 |
| Observational unit stay | 0.70 (0.31–1.58) | 0.40 |
| Chronic hemodialysis | 0.62 (0.33–1.16) | 0.14 |
| Surgery | 0.84 (0.50–1.41) | 0.51 |
| **Prior medication exposures^a^** |  |  |
| Antibiotic | 0.93 (0.66–1.32) | 0.69 |
| Proton pump inhibitor | 0.86 (0.62–1.19) | 0.36 |
| Immunosuppressant | 0.75 (0.52–1.07) | 0.11 |
| **CDI diagnosis and select clinical characteristics** |  |  |
| Hospital-onset CDI | 1.20 (0.59–2.44) | 0.61 |
| Diagnosis by a laboratory that provided no or   neutral comments | 3.15 (2.15–4.60) | <0.0001 |
| ≥3 unformed stools for ≥1 day | 1.95 (1.42–2.68) | <0.0001 |
| Hospital admission^b^ | 0.54 (0.34–0.86) | 0.01 |
| ICU admission^c^ | 1.12 (0.60–2.10) | 0.72 |
| WBC ≥15,000/µl | 1.85 (1.25–2.76) | 0.002 |

Abbreviations: NAAT, nucleic acid amplification test; CDI, *Clostridioides difficile* infection; ICU, intensive care unit; WBC, white blood cell

^a^During the 12 weeks prior to CDI diagnosis.

^b^Hospitalized at the time of or within 6 days following CDI diagnosis.

^c^Admitted to the ICU on the day of or within 6 days following CDI diagnosis.
